# Long-term outcomes after close rectal dissection and total mesorectal excision in ileal pouch-anal anastomosis for ulcerative colitis

**DOI:** 10.1007/s10151-022-02713-x

**Published:** 2022-11-06

**Authors:** M. A. Reijntjes, D. C. de Jong, S. Bartels, E. M. Wessels, E. K. Bocharewicz, R. Hompes, C. J. Buskens, G. R. d’Haens, M. Duijvestein, W. A. Bemelman

**Affiliations:** 1 Department of Surgery, Amsterdam UMC, Location AMC, University of Amsterdam, Meibergdreef 9, 1105 AZ Amsterdam, The Netherlands; 2 Department of Gastroenterology, Amsterdam UMC, Location AMC, Amsterdam, The Netherlands; 3 Department of Gastroenterology, Radboudumc, Nijmegen, The Netherlands; 4 IBD Unit, Gastroenterology and Endoscopy, IRCCS Ospedale San Raffaele and University Vita-Salute San Raffaele, Milan, Italy

**Keywords:** Ulcerative colitis, Ileal pouch anal anastomosis (IPAA), Quality of life, Mesorectum

## Abstract

**Background:**

During ileal pouch-anal anastomosis (IPAA) surgery for ulcerative colitis (UC), rectal dissection can be performed via close rectal dissection (CRD) or in a total mesorectal excision plane (TME). Although CRD should protect autonomic nerve function, this technique may be more challenging than TME. The aim of this study was to compare long-term outcomes of patients undergoing CRD and TME.

**Methods:**

This single-centre retrospective cohort study included consecutive patients who underwent IPAA surgery for UC between January 2002 and October 2017. Primary outcomes were chronic pouch failure (PF) among patients who underwent CRD and TME and the association between CRD and developing chronic PF. Chronic PF was defined as a pouch-related complication occurring ≥ 3 months after primary IPAA surgery requiring redo pouch surgery, pouch excision or permanent defunctioning ileostomy. Secondary outcomes were risk factors and causes for chronic PF. Pouch function and quality of life were assessed via the Pouch dysfunction score and Cleveland global quality of life score.

**Results:**

Out of 289 patients (155 males, median age 37 years [interquartile range 26.5–45.5 years]), 128 underwent CRD. There was a shorter median postoperative follow-up for CRD patients than for TME patients (3.7 vs 10.9 years, *p* < 0.01). Chronic PF occurred in 6 (4.7%) CRD patients and 20 (12.4%) TME patients. The failure-free pouch survival rate 3 years after IPAA surgery was comparable among CRD and TME patients (96.1% vs. 93.5%, *p* = 0.5). CRD was a no predictor for developing chronic PF on univariate analyses (HR 0.7 CI-95 0.3–2.0, *p* = 0.54). A lower proportion of CRD patients developed chronic PF due to a septic cause (1% vs 6%, *p* = 0.03).

**Conclusions:**

Although differences in chronic PF among CRD and TME patients were not observed, a trend toward TME patients developing chronic pelvic sepsis was detected. Surgeons may consider performing CRD during IPAA surgery for UC.

## Introduction

Restorative proctocolectomy with subsequent ileal pouch-anal anastomosis (IPAA) is a treatment option for patients with refractory ulcerative colitis (UC), familial adenomatous polyposis (FAP) and UC-related dysplasia. Proctectomy with IPAA can be performed at the time of colectomy (1- or 2-stage surgery) or following subtotal colectomy in a second stage completion proctectomy (modified 2- or 3-stage surgery). Both IPAA construction and (completion) proctectomy are associated with serious short- and long-term postoperative morbidity, such as anastomotic leakage which occurs in 10–20% of patients, and chronic pouch failure (PF) which occurs in 5–10% of patients [1–4]. Innovations in surgical techniques have been developed to improve short- and long-term outcomes after IPAA surgery [5–7]. One of the many surgical challenges during ileoanal pouch surgery is the dissection of the rectum. Dissection in the total mesorectal excision (TME) plane, similar to a dissection for cancer, should follow an avascular embryological plane, but autonomic nerves are at risk. Damage may result in sexual and/or urinary dysfunction [8]. In our institution, surgeons performed a posterior TME, preserving the anterolateral mesorectum as per European Crohn’s and Colitis organisation (ECCO) recommendations until 2007 [9]. A technically more challenging, complete close rectal dissection (CRD) was introduced in 2007 to spare the autonomic nerves. During CRD, dissection is performed through a non-anatomical perimuscular plane, well away from the autonomic nerves. This leaves a smaller dead space in the pelvis postoperatively, which may reduce postoperative septic collections in the pelvis [10, 11]. However, there is a potential risk of haemorrhage from the mesorectal vessels.

These rectal dissection techniques have been compared in a randomized controlled trial assessing short-term postoperative morbidity [12]. Results demonstrated that CRD led to a lower severe complication rate and better short-term quality of life when compared to TME. CRD has been performed as default technique for UC in our centre ever since. However, to date, long-term data on quality of life, pouch function and chronic PF among patients who underwent TME and CRD have been reported. The aim of this study was to assess chronic PF rates and long-term failure-free pouch survival among patients who underwent (completion) proctectomy and IPAA surgery via CRD and TME in a tertiary referral centre for pouch surgery.

## Materials and methods

### Design and patients

In this retrospective cohort study, all consecutive adult patients who underwent IPAA for UC in the Amsterdam University Medical Centers (UMC), location Academic Medical Center (AMC), between January 2002 and October 2017 were included. This is a tertiary referral centre with experienced inflammatory bowel disease (IBD) surgeons. Patient data were stored in a database constructed by four of the authors (MR, DJ, EW and EB). Patients who underwent IPAA surgery with a histopathologic diagnosis or suspicion of FAP, Crohn's disease or IBD-undetermined in the colectomy resection specimen, and patients undergoing redo-pouch surgery were excluded. Patients with unspecified rectal dissection types were also excluded. The medical ethics board have assessed and deemed review unnecessary for this study. All participants provided informed consent via opt-out procedure. Reporting of the data adheres to the Strengthening the Reporting of Observational Studies in Epidemiology (STROBE) Statement.

### Procedures

#### IPAA

All IPAA procedures were performed by experienced colorectal surgeons. Surgical creation of an IPAA was performed during initial proctocolectomy (1- or 2-stage IPAA surgery), or at the time of completion proctectomy (modified 2- or 3-stage surgery). A 10 cm J (or modified J) ileal pouch was created via transanal or transabdominal (by laparotomy or laparoscopy) approach, or a combined approach. A pouch-anal anastomosis was routinely constructed with a circular stapler. A defunctioning ileostomy was created selectively, based on intraoperative findings (e.g. traction on pouch-anal anastomosis, positive air-leak test).

##### Proctectomy types

**Fig. 1:**
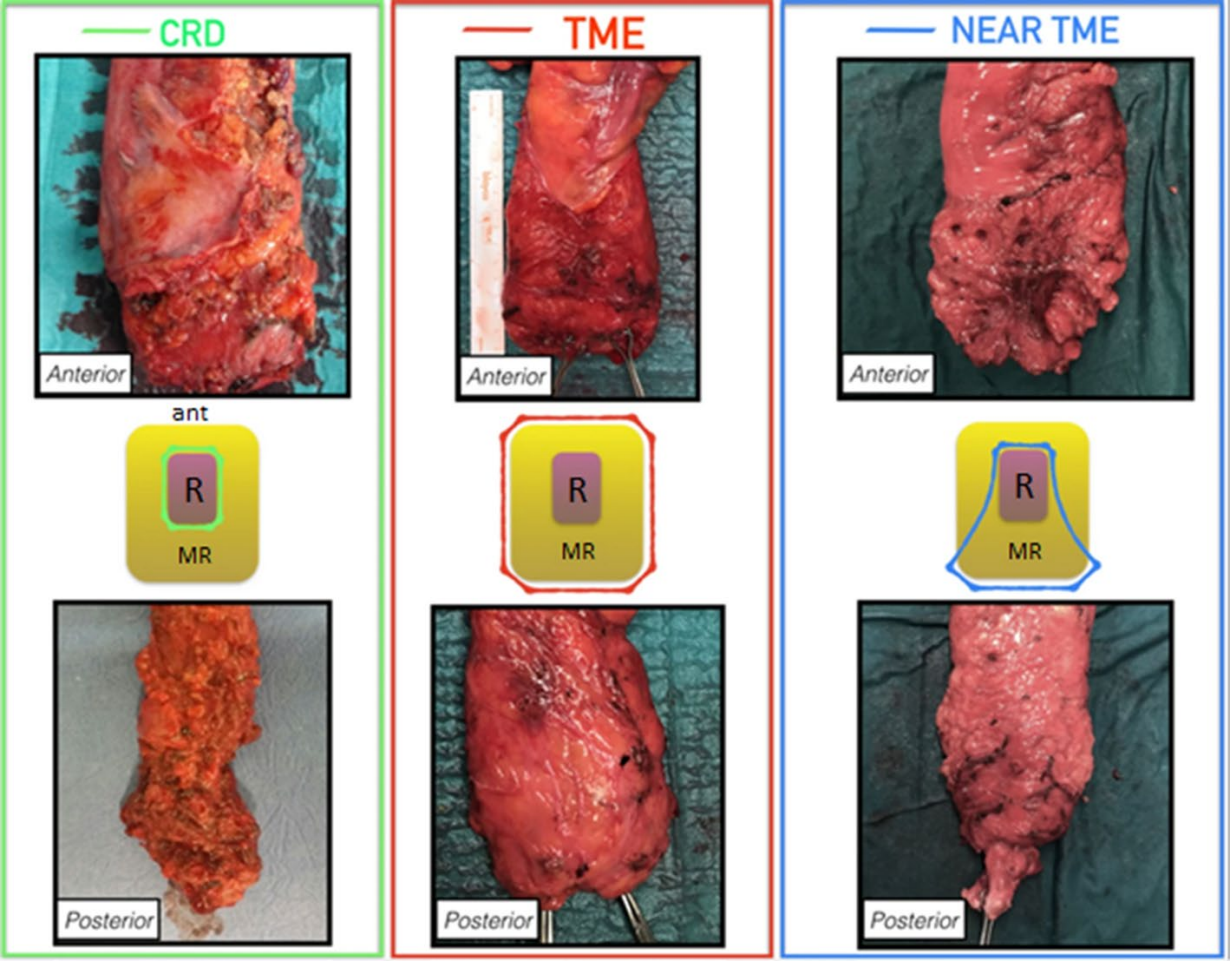
Resection specimens and schematic transversal resection margins for CRD (green), TME (red) and sub-TME (blue) dissection [11]. *TME* total mesorectal excision, *CRD* close rectal dissection, *Ant* anterior side of rectum, *R* rectum, *MR* mesorectal fat

Prior to the introduction of CRD as a dissection technique, a TME or ‘sub-TME’ was performed. The sub-TME was defined as an anterior rectal dissection performed on the outer muscle layer of the distal rectum with posterolateral mesorectal dissection. Subsequent to 2007, a CRD was routinely performed, leaving the mesorectum in situ by working in the non-anatomical perimuscular plane, sparing the superior rectal artery (Fig. 1) [11].

### Outcomes

Outcomes were compared for patients undergoing TME (including sub-TME) and CRD. Primary outcomes of this study were chronic PF and failure-free pouch survival at end of follow-up and 3 years after IPAA surgery. Secondary outcomes were risk factors and causes for chronic PF. Pouch function and quality of life were assessed via the Pouch dysfunction score (PDS) [13] (Appendix A) and Cleveland global quality of life (CGQL) score [14] (Appendix B).

### Definitions

Chronic PF was defined as a long-term (≥ 3 months) pouch-related complication (including pouch dysfunction) following initial IPAA surgery requiring pouch redo surgery or excision regardless of the surgical indication and functional outcome of the pouch (with or without permanent diversion by ileostomy). Chronic PF did not include redo-pouch surgery performed within 3 months postoperatively for acute postoperative complications. Follow-up was calculated from the first day of faecal stream through the pouch until the date of the last follow-up visit. In the current study, potential postoperative risk factors for chronic PF were assessed. Type of dissection, postoperative pelvic sepsis, and pouch fistula were included in the analyses. Pelvic sepsis included acute anastomotic leakage and/or chronic pelvic sepsis. A fistula that occurred after IPAA surgery originating from anywhere in the pouch was defined as a postoperative pouch fistula. Both pelvic sepsis and postoperative pouch fistula were diagnosed on postoperative cross-sectional imaging (computed tomography scan or magnetic resonance imaging).

### Statistical analysis

Categorical outcomes were expressed as percentage. Continuous levels of secondary outcomes showing were expressed as mean with standard deviation (SD) or median with interquartile range (IQR), according to the distribution. To compare categorical data, the Chi-square test was used for statistical significance. For comparing continuous data with normal distribution, the independent samples *T*-test was used. In case continuous data were not normally distributed, the Mann–Whitney *U* test was used to compare continuous levels. Uni- and multivariate Cox-regression analysis was used for analysis of failure-free pouch survival. CRD, pelvic sepsis and pouch fistula were included in the multivariate Cox-regression model. Pelvic sepsis and pouch fistula are established independent risk factors for chronic PF according to literature [4]. The final model was established by stepwise manual backward selection (*p* < 0.05 to stay in the model). Outcomes of the multivariate Cox-regression analysis are presented as hazard ratios (HR) with 95% confidence intervals (CI)*.* Survival analyses were performed via Kaplan–Meier analyses with log-rank as comparison test**.** Levels with missing data exceeding 10% of all cases were excluded from Cox-regression analyses. Agreement between fistula and pelvic sepsis was calculated by the Kappa measure of agreement test; agreement was slight, fair, moderate, substantial or perfect for 0.0–0.20, 0.21–0.40, 0.41–0.60, 0.61–0.80 and 0.81–1.00, respectively [15]. Data were analysed through complete case analyses. Differences were considered statistically significant if two-sided *p* values were below 0.05. Statistical analyses were performed using SPSS (IBM Corp., Armonk, NY, USA).

## Results

### Demographics

Ileoanal pouches were created in 341 UC patients between January 2002 and October 2017. A total of 52 patients were excluded from this study as there was a diagnosis other than UC (*n* = 29), the rectal dissection type remained unspecified (*n* = 20) or the IPAA never became functional (*n* = 3). The median age of the 289 included patients was 37.0 years (IQR 26.5–45.5 years), and 155 (53.6%) were male. Overall, 128 (44.3%) underwent proctectomy via CRD, and 161 (55.7%) patients underwent (sub)TME. Within the TME cohort, 54 (33.5%) patients underwent a sub-TME. Median age and sex were comparable among CRD and (sub)TME (35.0, IQR 25.8–45.3 vs. 38.0 IQR 27.5–46.0 and 69 (53.9%) vs 86 (53.4%) male, *p* = 0.17 and 0.93, respectively). The majority of the patients who underwent IPAA underwent proctectomy via a transabdominal approach (78.2%). A larger proportion of patients who underwent proctectomy via a CRD underwent IPAA surgery via a modified 2-stage IPAA surgery (78.9% vs. 41.0%, *p* < 0.01) and transanal proctectomy (46.1% vs. 1.2%, *p* < 0.01) when compared to patients who underwent (sub)TME. Further baseline characteristics are shown in Table 1.

**Table 1:** Baseline demographics

Patient characteristics	Total (*n* = 289)		CRD (*n* = 128)		(sub)TME (*n* = 161)			Missing	
	*n*	%	*n*	%	*n*	%	*p*-value	*n*	%
Male	155	53.6%	69	53.9%	86	53.4%	0.93	0	0.0%
Age at time of IPAA surgery, years, median(IQR)	37.0 (26.5–45.5)		35.0 (25.8–45.3)		38.0 (27.5–46.0)		0.17	0	0.0%
Staged surgery								0	0.0%
One-staged	50	17.3%	10	7.8%	40	24.8%	** < 0.01**		
Two-staged	34	11.8%	4	3.1%	30	18.6%			
Modified two-staged	167	57.8%	101	78.9%	66	41.0%			
Three-staged	38	13.1%	13	10.2%	25	15.5%			
Approach proctectomy								2	0.7%
Transabdominal	226	78.2%	68	53.1%	158	98.1%	** < 0.01**		
Transanal	61	21.1%	59	46.1%	2	1.2%			
Abdominal approach (*n* = 226)^a^								7	3.1%
Open	112	49.6%	24	35.3%	88	55.7%	** < 0.01**		
Laparoscopic/hand-assisted	107	47.3%	42	61.8%	65	41.1%			
Smoking									
Yes	33	11.4%	17	13.3%	16	9.9%	0.40	34	11.8%
Previously	23	8.0%	13	10.2%	10	6.2%			
No	199	68.9%	87	68.0%	112	69.6%			
ASA score									
1	54	18.6%	26	20.3%	28	17.4%	0.83	53	18.3%
2	172	59.3%	87	68.0%	85	52.8%			
3	10	3.4%	4	3.1%	6	3.7%			

*IPAA* Ileal pouch-anal anastomosis, *TME* total mesorectal excision, *CRD* close rectal dissection, *ASA* American Society of Anaesthesiologists, scored by an anaesthesiologist

^a^Patients who underwent transabdominal proctectomy

### Long-term outcomes after IPAA surgery

**Fig. 2:**
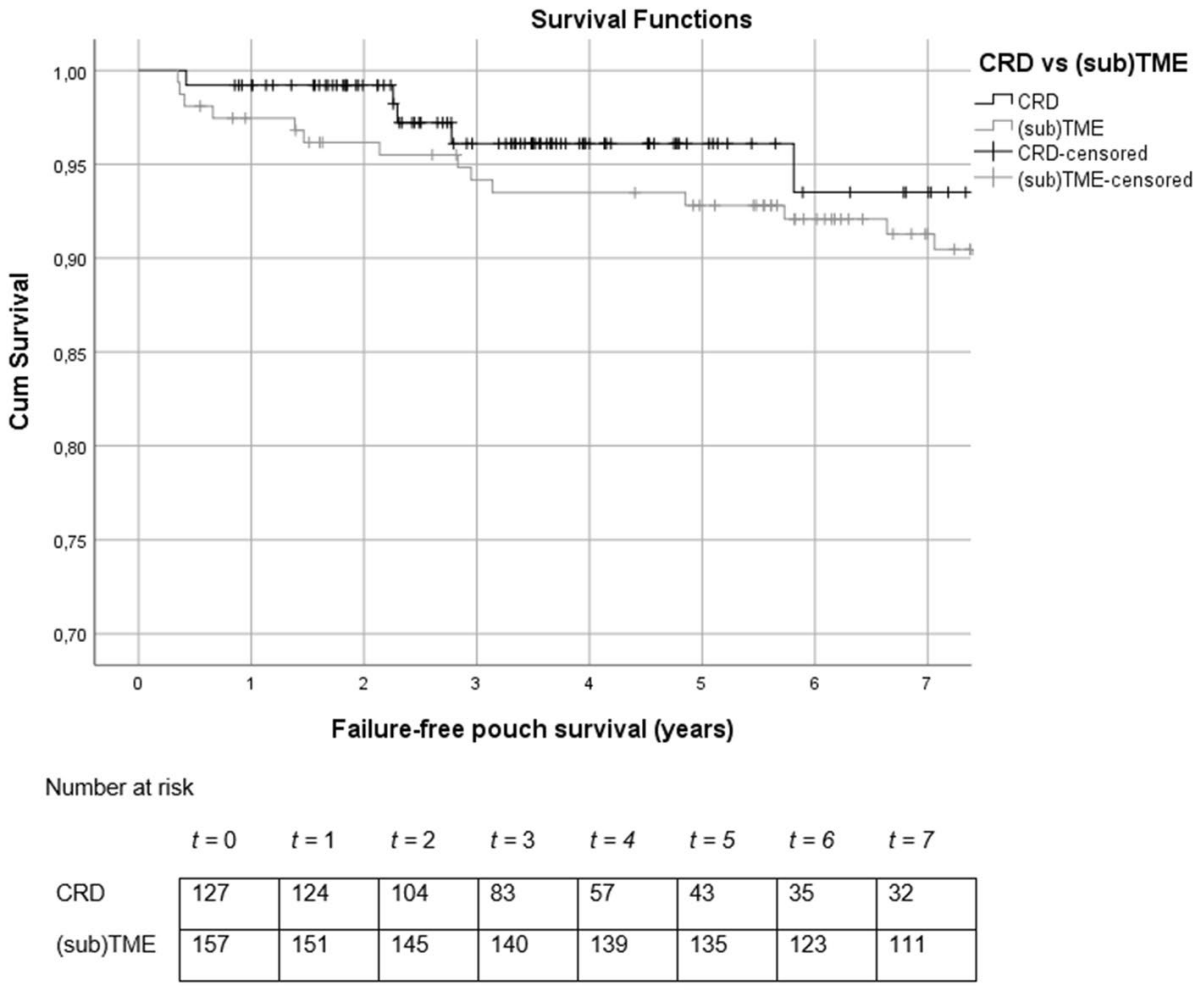
Failure-free pouch survival curve for patients who underwent CRD (black) and (sub) TME (grey). *TME* total mesorectal excision, *CRD* close rectal dissection, *Cum* cumulative

After a median follow-up of 7.2 (IQR 3.4–11.6) years, 26 out of 289 patients (9.0%) developed chronic PF. The median follow-up for patients with CRD was significantly shorter when compared to (sub)TME (3.7 vs. 10.9 years, p < 0.01). Chronic PF occurred in a smaller proportion of patients who underwent CRD when compared to patients who underwent (sub)TME (4.7% vs 12.4%, *p* = 0.02). Three (1.0%) patients were excluded from the survival analyses as they were lost to follow-up. Overall, mean chronic failure-free pouch survival was 17 (CI 16.2–17.8) years, and was comparable between patients who underwent CRD and (sub)TME (14.0 vs 16.8) years, *p* = 0.49). Failure-free pouch survival rates after 36 months were 96.1% for patients with a CRD dissection and 94.2% for patients with a (sub)TME dissection. Survival curves are shown in Fig. 2.

### Causes of chronic PF

Out of the six patients who developed chronic PF requiring surgery after CRD, one was a result of a chronic sinus. One patient developed chronic refractory pouchitis, and two patients developed outlet complications. Two patients underwent permanent defunctioning for dysfunction caused by high defecation frequency (*n* = 1) and incontinence (*n* = 1).

Out of 20 patients with chronic PF after (sub)TME, 10 had septic complications causing failure. Four patients developed pouchitis, 1 patient had inlet complications and 3 patients developed outlet complications. Two patients underwent permanent defunctioning for severe dysfunction due to incontinence (*n* = 1) and dysfunction after a radiation induced ulcer for prostate cancer (*n* = 1).

**Fig. 3:**
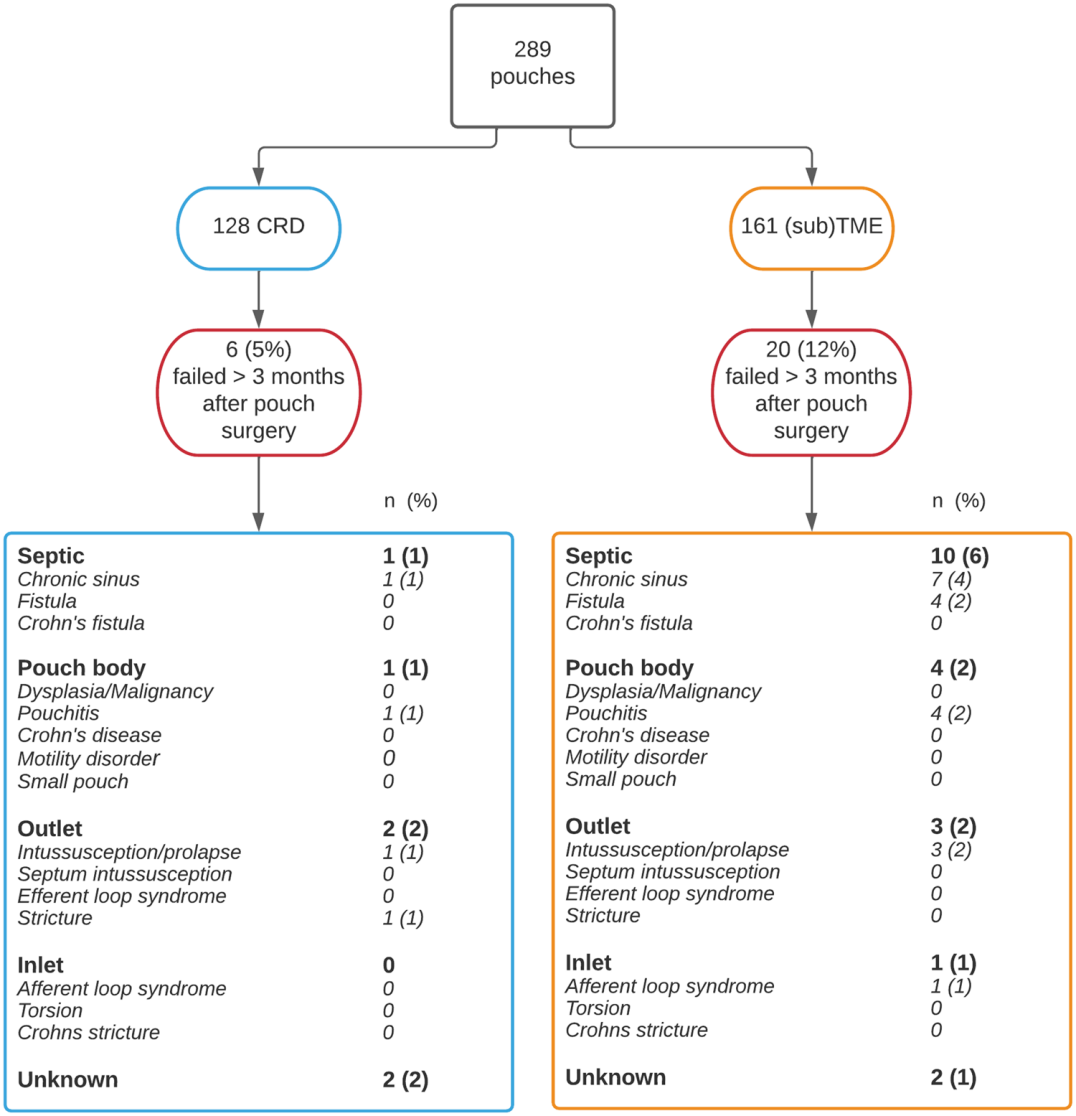
Causes of chronic PF in UC among patients underwent CRD and (sub)TME (*p* = 0.2). *PF* pouch failure, *UC* ulcerative colitis, *TME* total mesorectal excision, *CRD* close rectal dissection

**Fig. 4:**
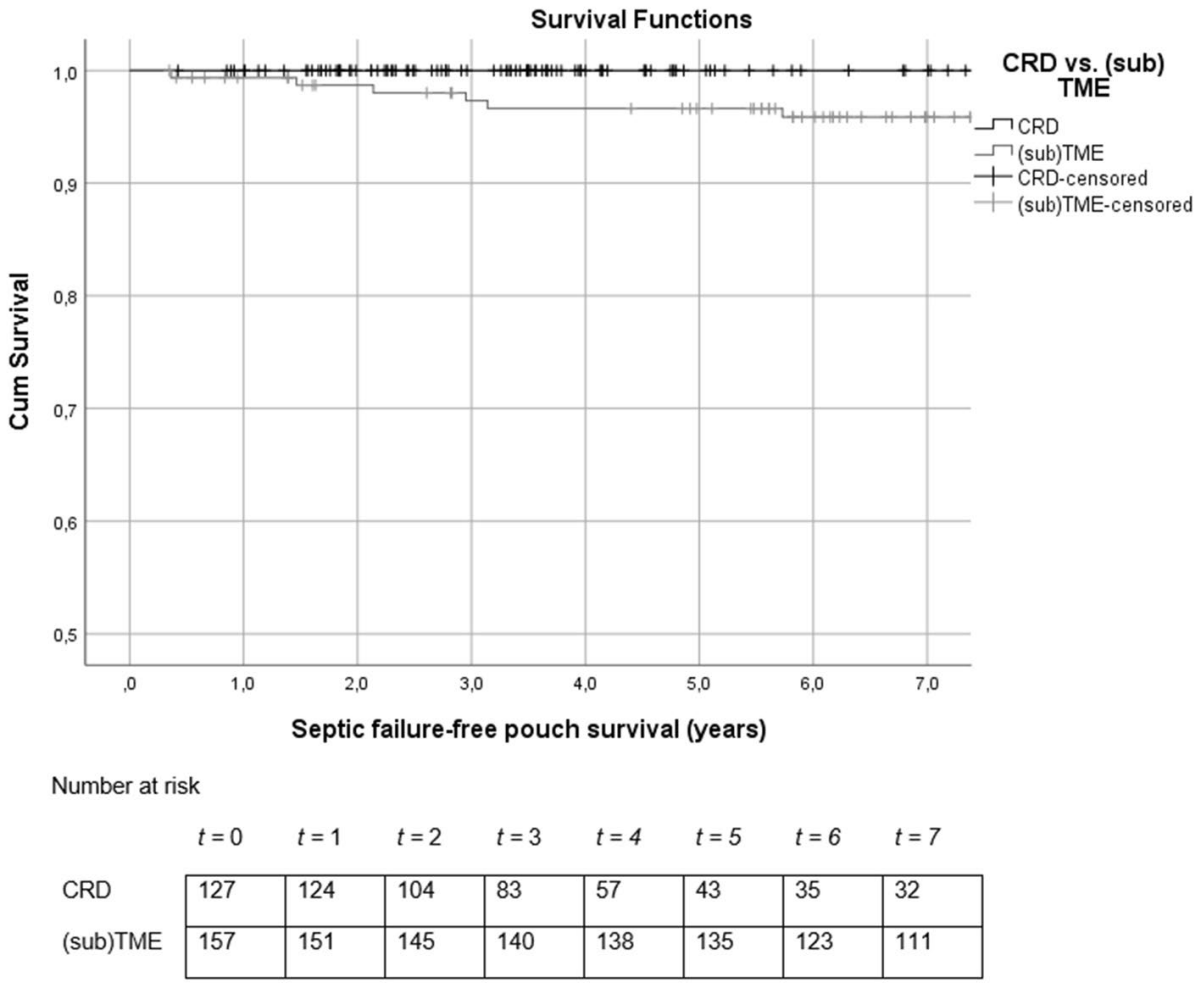
Survival curve of septic failure-free pouch survival among patients who underwent CRD and (sub)TME (*p* = 0.2). *TME* total mesorectal excision, *CRD* close rectal dissection, *Cum* cumulative

Figure 3 shows causes of chronic PF per rectal dissection technique. A significantly lower proportion of chronic PF caused by septic complications was found in CRD patients when compared to (sub)TME (0.8% vs 6.2%, *p* = 0.03). The septic failure-free survival was 100% in the CRD group compared to 96.6% in the (sub)TME group (log rank *p* = 0.2, Fig. 4).

### Risk factors for chronic PF

In this cohort, 42 patients (14.5%) developed pelvic sepsis. A chronic sinus not preceded by an established acute postoperative anastomotic leakage was diagnosed in 5 (1.7%) patients. Acute anastomotic leakage occurring within 90 days postoperatively was diagnosed in 37 (12.8%) patients, 19 14.8% in the CRD group and 18 (11.4%) in the (sub)TME group (*p* = 0.39). As of 2006, Endovac therapy was applied in 19 patients with acute anastomotic leakage, and combined with early surgical closure (EVASC) in 15 of these patients [16]. None of the patients who underwent CRD surgery followed by EVASC-treated acute anastomotic leakage developed chronic PF caused by septic complications (*n* = 12). Two out of three (sub)TME patients developed chronic PF caused by septic complications after EVASC therapy for anastomotic leakage. Moreover, 2 out of 4 (sub)TME patients with Endovac treatment alone developed chronic pelvic sepsis. Overall, 29 patients (10.0%) developed a pouch-related fistula. Univariate analyses revealed pelvic sepsis and pouch fistula as risk factors for developing chronic PF (HR 6.3, CI 95% 2.9–13.7, *p* < 0.001 and HR 7.1, CI 95% 3.3–15.4, *p* < 0.001, respectively). CRD was not a risk factor for developing chronic PF (HR 0.7 CI 0.3–1.9, *p* = 0.48). In multivariate analyses including pelvic sepsis, fistula and CRD, pelvic sepsis and fistula were independent risk factors for developing chronic PF (HR 3.3, *p* = 0.01 and HR 4.3, *p* < 0.01). A slight agreement was observed between pelvic sepsis and fistula (*κ*: 0.40). Results are shown in Table 2.

**Table 2:** Uni- and multivariate Cox-regression for failure-free pouch survival

Variable	HR	CI (95%)	*p*
Univariate Cox-regression			
Close rectal dissection (*n* = 128)	0.7	0.3–1.9	0.48
Pelvic sepsis (*n* = 42)	6.3	2.9–13.7	** < 0.001**
Pouch fistula (*n* = 29)	7.1	3.3–15.4	** < 0.001**
Multivariate Cox-regression			
Close rectal dissection (*n* = 128)	0.7	0.3–1.9	0.48
Pelvic sepsis (*n* = 42)	3.4	1.4–8.0	**0.006**
Pouch fistula (*n* = 29)	4.1	1.8–9.9	**0.001**
Multivariate Cox-regression			
Pelvic sepsis (*n* = 42)	3.3	1.4–7.8	**0.007**
Pouch fistula (*n* = 29)	4.3	1.8–10.3	**0.001**

The bold values indicates two sided significance of < 0.05 (they are significant risk factors for chronic PF) in either unior multivariable analyses

### Pouch function

**Fig. 5:**
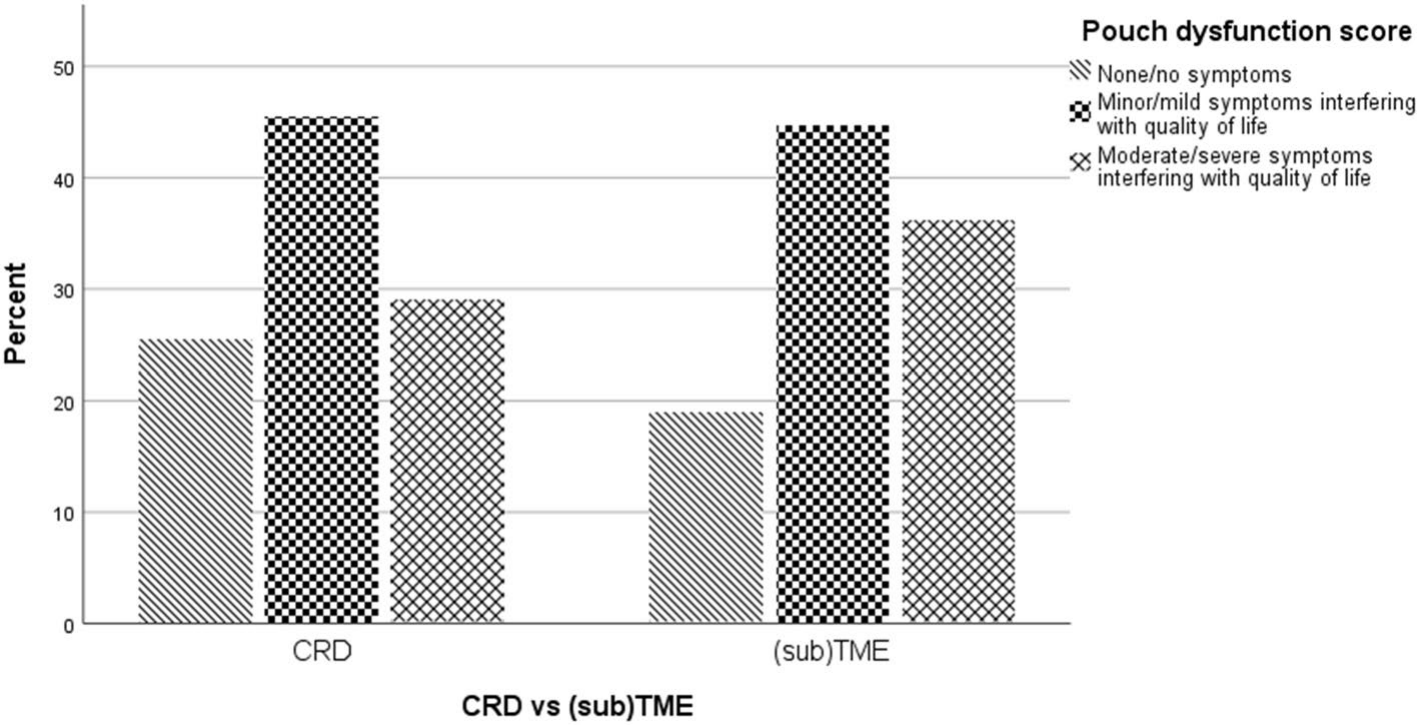
Bar chart representing the PDS for patients who underwent CRD (left) and (sub)TME (right)*. *TME* total mesorectal excision, *CRD* close rectal dissection, *PDS* Pouch dysfunction score, *PF* pouch failure. *Patients who developed chronic PF were excluded from pouch function analyses

After excluding 26 patients who developed chronic PF, the response rate for the pouch function questionnaires was 215/263 (81.7%). Follow-up was shorter for CRD patients compared to (sub)TME (31.5 months, IQR 16.0–51.3 vs. 126 months, IQR 78.8–150.8, *p* < 0.01). According to the PDS (Appendix A), 145 patients (67.4%) had ‘none to minor’ pouch dysfunction, and 70 patients (32.6%) had ‘some to major’ pouch dysfunction. When comparing 110 patients who underwent CRD with 106 patients who underwent (sub)TME, 32 (29.1%) of CRD patients had some/major dysfunction, compared to 38 (36.2%) of TME patients (*p* = 0.27). A lower proportion of CRD patients reported a defecation frequency exceeding ten times per 24 h, when compared to TME patients (21, 19.4% vs 32, 30.1%, *p* = 0.06). No difference in the continuous total PDS was reported between patients who underwent CRD and TME (1.5, IQR 0.4–2.5 vs. 1.5, IQR 0.5–2.9, *p* = 0.26) (see Fig. 5).  Quality of life according to the CGQL (Appendix B) was similar among patients who underwent CRD and TME (0.73 IQR 0.67–0.83 vs. 0.73 IQR 0.63–0.80, *p* = 0.32).

## Discussion

We found no significant differences in long-term outcomes between patients who had CRD during IPAA for UC and those who had (sub)TME. Failure-free pouch survival and functional outcomes were similar among cohorts and CRD was not associated with developing chronic PF. After a median follow-up of 7.2 years, an overall chronic PF rate of 9.0% was found in this study, which is in line with available literature [4]. Although chronic PF rates of CRD patients were significantly lower when compared to (sub)TME (4.7% vs. 12.4%, *p* < 0.01), this finding was confounded by a significantly shorter postoperative follow-up for patients who underwent CRD surgery (3.7 vs. 10.9 years, *p* < 0.01). No significant difference remained after correction for length of follow-up in survival analyses (*p* = 0.49). Pelvic sepsis and fistula were independent risk factors for chronic PF in the current study, which corresponds with the literature [4]. A larger proportion of patients who underwent CRD had a proctectomy via transanal minimally invasive surgery combined with a laparoscopic abdominal approach. Furthermore, a larger proportion of patients who underwent CRD underwent modified two -stage pouch surgery. These differences relate to the change in approach adopted by our unit over the period of the study. In the present study, there was a lower proportion of chronic PF secondary to a septic cause in CRD patients when compared to patients who underwent (sub)TME (0.8% vs. 6.2%). This is consistent with previous studies and perhaps supports the hypothesis that mesorectal fat preserved during proctectomy has a protective role against pelvic sepsis; less dead space remains in the pelvis postoperatively and both the lymphatic and nervous system are maintained [10, 17]. However, a potential confounder is the introduction of EVASC for acute anastomotic leakage in 2010, which may have also reduced pouch failure due to chronic pelvic sepsis. Nevertheless, 2 out of 3 (sub)TME patients with EVASC-treated acute anastomotic leakage developed chronic pelvic sepsis, whereas none of the 12 CRD patients did. Another potential confounder to our results is the introduction of a more intense postoperative diagnostic protocol introduced during the study period. Whilst the acute postoperative anastomotic leakage rates were similar among CRD and (sub)TME patients, we hypothesize that the diagnosis of acute postoperative leakage after (sub)TME may have been underestimated, resulting in an increased proportion of chronic septic complications. A chronic sinus generally does not appear de novo after absence of acute postoperative complications, as it is most likely a product of (un)diagnosed acute postoperative anastomotic leakage [5, 18, 19]. Undiagnosed postoperative leakage may have been less common after CRD surgery because this intensive follow up protocol was introduced around the same time. The increased incidence of PF caused by septic complications after (sub)TME surgery could therefore, to a lesser extent, be due to the difference in postoperative follow-up.

To the best of our knowledge, this is the first study assessing long-term outcomes after CRD and (sub)TME proctectomy during IPAA surgery. This is a large cohort of 289 patients with a median follow-up period exceeding seven years. Although comorbidities were not reported, ASA scores representing overall preoperative condition were collected and similar among cohorts. Therefore, this study attempted to take potential confounding risk factors for chronic PF into account. Nevertheless the study has some limitations in addition to the potential innovation confounders discussed above. Although the retrospective nature of this study may have resulted in missing data on baseline characteristics and follow-up, pouch complications were, by default, diagnosed and (surgically) treated in our referral centre. Therefore, the follow-up is likely to be complete. We assessed pouch function and quality of life via validated questionnaires. Pouch function and quality of life were comparable among cohorts, with a slightly lower proportion of CRD patients who experienced a high frequency of defecation. However, conclusions cannot be adequately drawn based on these results due to the difference in follow-up time. One potential benefit of a CRD approach is the hypothesis that this technique might spare the autonomic nerves involved in sexual and urinary functions. Earlier studies have not reported significant differences in sexual function among patients who underwent CRD and TME [22, 23]. In the current study, we did not record sexual and urinary function, as these were not routinely assessed during postoperative outpatient visits. Furthermore, the shorter postoperative follow-up period in CRD patients preclude adequate comparison of data on sexual and urinary function. Potential major confounding factors in terms of surgical technique (e.g. number of stages, laparoscopic or open abdominal approach) were not included in multivariate analyses due to a limited number of chronic PF events (*n* = 26). Two risk factors for chronic PF (pelvic sepsis and pouch fistula), previously established by a meta-analysis, were included in our multivariate analyses. Although pouch fistula and pelvic sepsis were both independent risk factors for chronic PF, these complications occasionally occur concurrently and a causal relation between anastomotic leakage and developing fistula may reasonably be inferred. However, only a slight level of agreement between these variables was found. Moreover, this correlation should not have interfered with (multivariable) analyses since CRD was not associated with chronic PF in analyses.

## Conclusions

Chronic failure-free pouch survival rates were comparable among cohorts and CRD was not an (independent) risk factor for chronic PF. Patients who underwent CRD had a lower rate of chronic PF caused by pelvic sepsis, possibly due to decreased postoperative dead space in the pelvis after CRD surgery and implementation of EVASC. Although future studies evaluating long-term pouch function and quality of life are warranted, results of this study demonstrate at least similar long-term outcomes after CRD when compared to (sub)TME.
